# Australian Food Safety Policy Changes from a “Command and Control” to an “Outcomes-Based” Approach: Reflection on the Effectiveness of Its Implementation

**DOI:** 10.3390/ijerph13121218

**Published:** 2016-12-08

**Authors:** James Smith, Kirstin Ross, Harriet Whiley

**Affiliations:** Health and the Environment, School of the Environment, Flinders University, GPO Box 2100, Adelaide 5001, Australia; jim@publichealthplanning.com.au (J.S.); Kirstin.Ross@flinders.edu.au (K.R.)

**Keywords:** food safety, regulatory approach, command and control

## Abstract

Foodborne illness is a global public health burden. Over the past decade in Australia, despite advances in microbiological detection and control methods, there has been an increase in the incidence of foodborne illness. Therefore improvements in the regulation and implementation of food safety policy are crucial for protecting public health. In 2000, Australia established a national food safety regulatory system, which included the adoption of a mandatory set of food safety standards. These were in line with international standards and moved away from a “command and control” regulatory approach to an “outcomes-based” approach using risk assessment. The aim was to achieve national consistency and reduce foodborne illness without unnecessarily burdening businesses. Evidence demonstrates that a risk based approach provides better protection for consumers; however, sixteen years after the adoption of the new approach, the rates of food borne illness are still increasing. Currently, food businesses are responsible for producing safe food and regulatory bodies are responsible for ensuring legislative controls are met. Therefore there is co-regulatory responsibility and liability and implementation strategies need to reflect this. This analysis explores the challenges facing food regulation in Australia and explores the rationale and evidence in support of this new regulatory approach.

## 1. Introduction

Worldwide, foodborne illness is a major public health issue [1,2]. Havelaar et al. [3] estimated that the global burden of foodborne illness was 33 million Disability Adjusted Life Years (DALYs). It is difficult to determine the true incidence of foodborne illness due to variation in reporting methods and a large number of cases going unreported [1,4]. Kirk et al. [5] estimated that in Australia during 2010 there was 4.1 million cases of foodborne illness. Of these there were 30,840 foodborne gastroenteritis-associated hospitalizations and 76 deaths. These estimates are based on data collected from notifiable disease surveillance at the national and state levels through the OzFoodNet; the National Gastroenteritis Survey II; and the Water Quality Study. It was determined that foodborne illness was responsible for approximately 25% of all gastroenteritis cases in Australia. In 2011, OzFoodNet reported that there were a total of 151 outbreaks of foodborne illness in Australia which resulted in 2104 confirmed cases, 231 hospitalisations and five deaths. Of these 56% were linked to restaurants, takeaways, commercial caterers or bakeries and 18% were linked to aged care facilities or hospitals [6]. Kirk et al. stated that only 9% of foodborne outbreaks in 2010 could be attributed to food preparation in private dwellings. Olsen et al. [7] demonstrated that the most commonly identified factors resulting in foodborne outbreaks include: improper holding and cooling of foods, poor personal hygiene of food handlers, cross-contamination, inadequate cooking and unsafe food sources.

It follows that the development of an appropriate food safety policy and its effective implementation in the hospitality and retail sector is a public health priority.

## 2. Command and Control versus an Outcomes-Based Food Policy

The command and control policy approach utilises a set of prescriptive requirements which detail well defined standards with which a food business must comply [8]. These regulatory requirements are often driven by the government, without allowing for flexibility or ingenuity from food business owners [9]. The hallmarks of a command and control approach to food safety consist of practices associated with inspections and end-product testing [10,11,12] which by their nature are reactive rather than preventative in that inspections identify, and end product testing confirms, food safety failures. This rules-based regulatory approach alone will therefore not prevent food borne illness.

However, the outcomes base approach requires food businesses to conduct a risk assessment in order to identify and control hazards [8]. It has been argued that an outcomes-based approach allows for creativity and innovation by industry in a way not possible with a command and control approach [13]. The outcomes-based approach allows for self, or cooperative regulation, which may better achieve goals than deterrence policies and adversarial strategies [13,14]. The differences between command and control and outcomes based policy is shown in Table 1.

## 3. Food Policy Regulation

In command and control approaches, standards and targets are set by government. However, the responsibilities for food safety were assumed to be the responsibility of the regulator as they had to ensure standards and targets were met. Aalders and Wilthagen [13] argue that direct regulation has had limited success in dealing with occupational health and safety, and with environmental regulation. A similar view was reached by Powell et al. [15] in relation to food safety where a culture of food safety, built on shared values, has been demonstrated to provide a far more effective approach to food safety compared with an inspectorial approach. This contrasts with an enforced self-regulation approach, where there is a mix of efforts between businesses and regulator, with businesses required to meet government set standards and to deal with non-compliance with regulators overseeing these efforts and, if necessary, imposing sanctions [16]. The success of the co-regulatory model pivots around the commitment and capacity of businesses to self-regulate and the ability of the regulator to find and maintain an optimal monitoring and oversight role [16].

From a food safety policy perspective, outcomes-based regulation, with a focus on safe and suitable food, can be viewed as a risk based approach. According to Hutter and Amodu [16] risk based regimes are more manageable and controllable and tend to be anticipatory, that is, focused on the prevention of risks and thus lead to an emphasis on output rather than prescriptive regulation. Martinez [17] argues that risk-based approaches to food safety regulation seek to ensure that greater emphasis is placed on food business operators managing their risks, resulting in a more targeted enforcement action and thereby reducing the regulatory burden. However, there are a number of issues that may affect the success of an outcomes-based approach. One of the biggest issues is food handlers’ lacking the required expertise and knowledge [8]. Taylor [18] identified that this was also one of major issues limited the success of the HACCP system within small food businesses in the UK. These challenges were identified by Yapp and Fairman [8] who explored the factors affecting small and medium sized food businesses compliance with food safety legislation in the UK. During this study the Food Safety Act 1990 was the primary Act governing food safety and was based around both prescriptive command and control requirements and self-regulatory approaches. Yapp and Fairman [8] found that the most common factors preventing compliance included lack of time and money, distrust of the food safety legislation and enforcers, lack of motivation and lack of knowledge and understanding.

## 4. Global Trends in Food Policy

Globally, food policy is constantly evolving to deal with new challenges, including changes in food safety hazards as a result of global food trade and the emergence of new risks such as genetically modified organisms [19]. It has also evolved in response to political pressures and a desire to increase consumer confidence [20]. In other disciplines, such as conservation, environmental protection, and occupational safety there has been attempts to move away from “command and control” to an outcomes-based regulatory approach [13]. Occupational health and safety has developed an approach whereby governments regulate self-regulation [21]. This approach encourages organisations to establish processes to monitor, control and replace activities that might be harmful to health and safety, and attempts to create a culture of responsibility for safety within the organisation [21]. Environmental protection has also attempted to move away from command and control to a risk based approach although, in contrast with the occupational health and safety approach, it has been primarily to introduce alternatives such as effluent taxes and marketable pollution permits [22].

Across Europe there has been a widespread support of risk based or outcomes-based regulation as mechanism for improving the quality and efficiency or governance [23]. In the UK specifically, it has been observed that governments over the past two decades have promoted the outcomes- based regulatory approach. However, they have found that outcomes-based approach to food safety regulation a difficult premise due to the governance issues of not producing unsafe food and the ideal of an acceptable level of risk [24]. In 2006, The European Union ratified new food hygiene regulations which shifted away from the command and control approach to a self-regulatory outcomes-based approach. The new policy placed the responsibility with the food business owners to demonstrate that they had controls in placed which effectively managed the food safety risks associated with their business [23]. In Australia any risk associated with producing unsafe food is considered unacceptable especially with regards to vulnerable groups [25].

## 5. Australian Changes in Food Policy

In 2000, Australia developed a national approach to food safety policy, rather than each state and territory developing its own food safety policy and legislation. Much work took place to develop a national food safety policy framework, including a set of food standards [10,26,27]. The aim of the policy was to provide a regulatory approach that would reduce the incidence of food borne illness and provides legislation that clearly outlines food safety obligations of both regulators and businesses [28]. The move to a national regulation making processes managed by a national food authority was seen to be beneficial due to economies of scale and resultant savings from this and to reduce cross-jurisdictional discrepancies in regulations [29] (Office of regulation review, 1995). It was recognised that the proposed uniformity may be problematic due to the inflexibility of national regulations to sufficiently deal with differences in local conditions [29].

The development of a Model Food Act based on this regulatory policy had its provisions adopted by states and territories with the result that the legislative objects of each state and territory’s food safety legislation is safe and suitable food. Although each state and territory have general food safety provisions contained within their respective acts of parliament, there are also quite specific, or prescriptive, provisions contained in the mandatory Food Standards Code adopted by each state and territory.

The Australian food safety policy makers decided to utilise the outcomes-based approach [28,30,31,32]. The argument for the move to an outcomes-based approach was based on both public health and economic arguments. The public health argument was that the prescriptive approach was not truly preventative and thus, the costs of failure were increasing the incidence and cost of food borne illness [30]. The move from prescription to outcomes-based regulation in food safety was consistent with a set of good regulatory practice principles endorsed in 1995 by the Council of Australian Governments (COAG) requiring that regulatory measures reflect the minimum required to achieved desirable outcomes and should be assessed using a scientifically robust risk assessment processes [10]. Subsequently, on 3 November 2000 the COAG agreed to major changes in food regulation including the establishment of the Australia New Zealand Food Standards Council, National Food Authority, and development of an intergovernmental food regulation agreement between the states and territories [10,30]. As Smith [30] noted the most significant part of these changes was a change in policy approach from what is referred to as a “command and control” approach to an outcomes-based approach. The National Food Authority (NFA) and Australia New Zealand Food Authority (ANZFA), the predecessors to Food Standards Australia New Zealand (FSANZ), argued that these prescriptive food safety activities needed to be replaced with goal, or outcome orientated legislation, as had been seen in a number of other countries [31,32].

The Food Regulation Agreement between the Commonwealth and states/territories resulted in the adoption of the Food Standards Code into the legislative framework of each state and territory. For the first time states and territories adopted the same food safety standards through their respective Acts of Parliament. Of specific interest to this discussion is the development and implementation of Chapter 3 of the Food Standards Code adopted into the Food Standards Code in 2000 and consisting of three food safety standards: Standard 3.1.1 (Interpretation and application; Standard 3.2.2 (Food safety practices and general requirements); and Standard 3.2.3 (Food premises and equipment). These standards constitute the common food safety legislative requirements for owners of food businesses throughout Australia and, subsequently provide the basis for the interaction between local regulators and food business owners.

The philosophy underpinning the development of the food hygiene reforms consisted of the following six principles:

1. partnership with stakeholders
2. consistency and clarity
3. risk-based minimum appropriate regulations
4. flexibility and practicality
5. preventative and outcomes focused regulations; and
6. international alignment [32].

Regulatory impact analysis concluded that the proposed standards represented the best way to:

- reduce the incidence of foodborne illness in Australia;
- reduce the incidence of foodborne contamination reaching the marketplace rather than detecting it after it has happened;
- encourage a business environment to respond quickly to emerging foodborne pathogens; encourage a business environment to take full responsibility for the safety of food produced; and
- support export initiatives to enable Australia to compete more effectively on world food markets [26].

According to Martin et al. [26] the above outcome-based food safety standards shifted the responsibility for food safety to the individual food business. The authors also stated that the standards would move businesses towards a preventative approach to managing food safety risks within their business. Undoubtedly this was the intention of policy makers but it cannot be assumed that this preventative focus will be achieved simply through the formulation of new mandatory standards.

## 6. Australian Implementation of the Outcomes-Based Approach to Food Safety Regulation

In addition to an appropriate policy approach there is a need for effective implementation. For the purposes of this discussion, policy implementation is concerned with those actions and factors put into effect to secure policy objectives [33], in this case, safe and suitable food. The implementation of the new food safety regulatory policy is the responsibility of the six states and two territories (Northern Territory and Australian Capital Territory) and the administration and enforcement of food hygiene legislation, contained in the Food Safety Standard (Chapter 3) of the Food Standards Code, at the retail level is undertaken generally by local council environmental health officers (EHOs) [31], or other authorised officers. As the Food Safety Standard is concerned with controlling the common causes of food borne illness, it is critical from the policy implementation perspective that these officers undertake an approach to food safety regulatory practices that is consistent with an outcomes and prevention based approach, which underpins the national food safety regulatory policy.

In Australia implementation lies primarily with a level of the government that is not party to policy development. Constitutional responsibility for food regulation lies with the states with local councils playing a key role in the administration and enforcement of consumer food safety regulation, except in the Australian territories [28]. The administration and enforcement at the retail level of food hygiene standards contained in the Food Safety Standard (Chapter 3) of the Food Standards Code is undertaken largely by local council environmental health officers (EHOs). Although the Standard has been adopted into the food safety legislative framework of each state and in Northern and Australian Capital territories, their administration and enforcement is undertaken by local councils. In 1995 there were over 600 agencies throughout Australia having responsibility for domestic food regulation [29]. The coordination of a large number of local agencies involved in food regulation and the development of guidelines at the federal level for adoption and consistent implementation by local regulators presents a challenging complexity to the implementation of the Standard across Australia. This task of coordinating implementation and enforcement of the new standard was recognised by policy makers as the 2000 Food Regulation Agreement also established an implementation framework consisting of the Australia and New Zealand Food Regulation Ministerial Council (Ministerial Council) and a Food Regulation Standing Committee (FRSC) with the Committee having responsibility for ensuring a consistent approach to the enforcement of the food standards. In addition, an Implementation Sub Committee (ISC) of FRSC was established with the role to ensure a consistent approach to the implementation and enforcement of food regulations and standards [34]. To that end a food regulation enforcement guideline was developed with the last revision in October 2015, and a document outlining the principles for the inspection of food businesses was developed in 2015 by the ISC [35]. The former document, although no doubt important in assisting in consistent application of legislative sanctions, is focussed on the consequences of non-compliance with the standards rather than focussing on food safety preventative measures. The purpose of the latter document is to provide local government EHOs with principles to guide inspections of food businesses to ensure the management of food safety risks and comply with the relevant food safety standards [35]. The aim of the document clearly focuses on food safety risks and the food safety performance of businesses which is consistent with the adopted outcomes-based regulatory approach. However, the document’s first page contains a disclaimer which states that the information presented as an information source only and that the reader, presumably a local council officer, is responsible for making their own assessment of matters and are advised to verify all relevant information presented [35]. This raises questions as to the status of such a document as it seems to be a heavily qualified advisory that does not appear to be consistent with the role of “overseeing” a consistent approach on implementation across jurisdictions. Given that there is no formal recognition of local government under the Australian Constitution could it be that the role of the ISC has been overstated? Perhaps the overseeing role is more relevant to state and territory governments that were the signatories to the Food Regulation Agreement rather than to local government which wasn’t a signatory. It is unclear as to what the implementation mechanisms are for the ISC across all jurisdictions responsible for implementation of the food safety standard. These mechanisms are considered critical for the performance of its currently stated role.

Local food safety regulatory resources has been a further constraint to the implementation of the standard and was recognised in 1995, well before the development of the food safety standard, by the Officer of Regulation Review (ORR) [29]. The ORR noted that resources for administration and enforcement of food legislation was an issue as the majority of agencies surveyed had indicated that although it was their policy to enforce all legislative provisions however, around half indicated that they were unable to do so in practice. The ORR also noted that there was no pattern to the resourcing local government agencies as, being autonomous, councils resourced their health units as they saw fit [29]. In 1999 the Australia New Zealand Food Authority (ANZFA) referred to this finding and estimated that enforcement of the food hygiene regulations would require and extra 250 full time EHOs at a cost of around $12 million [36].

The difficulty for local councils is that they have delegated responsibilities under each states and territories’ legislation and these delegations and are unlikely to involve a resourcing component. The Productivity Commission has identified that in recent year a main concern of local governments is “cost shifting” by the states onto local government. It has been identified that many local governments do not have sufficient resources to effectively undertake their regulatory functions, both in terms of finances and appropriately skilled staff [37].

The issue of a lack of resources at the local level had been identified prior to the development of the food safety standard and again in the 2012 by the Productivity Commission twelve years after the implementation of the Standard. These on the ground resources are a critical factor for the implementation of the Food Safety Standard as the Standard demands a specialised resource at the local level, that is, officers that have been trained in adapting new risk based approach methods to food safety rather than using the command and control methods of inspections and end product testing. These officers, generally environmental health officers, are university qualified in the application of risk principles to food safety; however, there are issues in practice and skill development in a command and control policy environment demanding compliance with prescribed standards. If the outcome required is safe and suitable food and there is a requirement to move from a command and control to an outcomes-based approach then there is a clear implication that regulatory practices need to orientate to prevention and this, in turn, requires the identification, targeting and management of food process risks in the food business [10]. This also a requires a change in philosophy regarding the inspectors traditional regulatory role and instead place emphasis on identifying risks that are likely to lead to foodborne disease [12].

Clearly the food safety role of local government regulators firstly is to use risk analysis to identify risk management priorities within business, and secondly check the effectiveness of control measures implemented by businesses and assess legislative compliance [10,17]. Initially, no additional training or resources was provided during the implementation of the change in food regulation; however, this gap is now being recognised in Victoria and EHOs are now receiving additional training on conducting risk based food inspections.

## 7. Evaluating the Outcomes of the Change in Policy

In Australia, the change in food safety policy from a “command and control” approach to an “outcomes-based” approach has not successfully decreased the incidence of foodborne illness. In fact, in 2000 the total incidence rate of notified gastrointestinal illness was 149.2 per 10,000, which has increased to 190.8 per 100,000 population in 2015 [38]. This equates to 44,764 cases of notified gastrointestinal illness [38] and using the estimate from Kirk et al. [5], then approximately 25% (11,191) of these notified cases can be attributed to foodborne illness. Notably, Kirk et al. [5] estimate that that the overall incidence of foodborne illness in Australia has recently declined, but identified that it is still a significant public health burden with listeriosis and salmonellosis the leading causes of foodborne associated death. Over the last decade the incidence of salmonellosis has significantly increased from 36 notified cases per 100,000 population in 2000 to 71.5 per 100,000 in 2015 [39]. Reports from OzFoodnet, also demonstrate that the total number of foodborne outbreaks in Australia, excluding those from private residence, has also increased over the past decade, from 81 in 2000 to 105 in 2013 [40,41,42,43,44,45,46,47]. This observed increase in foodborne illness may be influenced by global food trade [48] as well as increased intensity of farming [49]. However, one of the biggest factors influencing food borne outbreaks is improper food handling practices [50]. As such, the increase in incidence of food borne illness as a result of poor food handling practices might possibly be caused by poor implementation of changed policy rather than a failure of the outcomes-based approach to food safety regulation.

## 8. Suggested Changes to the Policy Implementation Process

Future studies are required to determine the effectiveness of different implementation approaches. Some areas warranting investigation include:

- Evaluating the risk assessment models and identification of critical control point(s) in food premises (especially small and medium sized food premises)
- Evaluate the food inspection process and communication between food business owners and Environmental Health Officers
- Evaluate the effectiveness and availability of food safety education programs
- Evaluate the availability of consultancy expertise (especially for small and medium sized food premises)
- Evaluate the mechanisms for notifying foodborne outbreaks and increased collaboration between human and veterinary public health professionals.

## 9. Conclusions

The outcomes measure of the current Australian food safety regulatory policy is the reduction in incidence of foodborne illness. However, sixteen years after the adoption of the new food safety regulatory policy there has been no observed decrease. This could be attributed to a range of factors such as changes in food hazards as a result of global food trade or increased farming. However it is likely that a significant proportion could be attributed to failures of food handling practices. This is despite the adopted food safety regulatory policy approach, beginning with the development of a national regulatory framework, seeming to be the most appropriate approach. The difficulty lies in the implementation of such a policy approach, which is dependent on local councils for most of the implementation, within Australia’s federated government structure. The level of implementation planning cannot be underestimated in such a structure and it demands robust communication, support, monitoring and feedback processes between policy formulators and policy implementers and a thoughtful consideration of not only the required resources for implementation but also the policy demands significant changes in long standing processes and practices at the local level. It is well recognised within Australia that national policy is not easily implemented however this commentary underscores the requirement for careful policy implementation and consideration of appropriate non-legislative change management strategies.

## Figures and Tables

**Table 1:** Policy overview of command and control approach versus outcomes-based approach.

	Command and Control	Outcomes-Based
Policy intent	The approach utilises detailed and prescriptive requirements by the government which must be followed by all designated food businesses i.e., one size fits all. The measured outcome is compliance with requirements.	The approach utilises risk assessment principles to identify hazards and control measures specific to individual food businesses. The measured outcome is the reduction in food borne illness.
Implementation	Checklists, interpretation guidelines, end product sampling protocols, and required inspections by regulators of all food businesses.	Individual food premises conduct risk assessments and identify “control points” to prevent foodborne illness and regulators assess the performance of these controls (cooperative regulation).
